# Comparative analysis of the activation of unfolded protein response by spike proteins of severe acute respiratory syndrome coronavirus and human coronavirus HKU1

**DOI:** 10.1186/2045-3701-4-3

**Published:** 2014-01-13

**Authors:** Kam-Leung Siu, Ching-Ping Chan, Kin-Hang Kok, Patrick C-Y Woo, Dong-Yan Jin

**Affiliations:** 1 Department of Biochemistry, The University of Hong Kong, 3/F Laboratory Block, Faculty of Medicine Building, 21 Sassoon Road, Pokfulam, Hong Kong; 2 Department of Microbiology, The University of Hong Kong, Pokfulam, Hong Kong

**Keywords:** SARS coronavirus, Human coronavirus HKU1, Spike, ER stress, Unfolded protein response

## Abstract

**Background:**

Whereas severe acute respiratory syndrome (SARS) coronavirus (SARS-CoV) is associated with severe disease, human coronavirus HKU1 (HCoV-HKU1) commonly circulates in the human populations causing generally milder illness. Spike (S) protein of SARS-CoV activates the unfolded protein response (UPR). It is not understood whether HCoV-HKU1 S protein has similar activity. In addition, the UPR-activating domain in SARS-CoV S protein remains to be identified.

**Results:**

In this study we compared S proteins of SARS-CoV and HCoV-HKU1 for their ability to activate the UPR. Both S proteins were found in the endoplasmic reticulum. Transmembrane serine protease TMPRSS2 catalyzed the cleavage of SARS-CoV S protein, but not the counterpart in HCoV-HKU1. Both S proteins showed a similar pattern of UPR-activating activity. Through PERK kinase they activated the transcription of UPR effector genes such as Grp78, Grp94 and CHOP. N-linked glycosylation was not required for the activation of the UPR by S proteins. S1 subunit of SARS-CoV but not its counterpart in HCoV-HKU1 was capable of activating the UPR. A central region (amino acids 201–400) of SARS-CoV S1 was required for this activity.

**Conclusions:**

SARS-CoV and HCoV-HKU1 S proteins use distinct UPR-activating domains to exert the same modulatory effects on UPR signaling.

## Introduction

Coronaviruses are enveloped viruses with a long positive-stranded RNA genome of ~30 kb. Their replication occurs in the cytoplasm and has a profound impact on the endoplasmic reticulum (ER) [1,2]. Particularly, extraordinarily large amounts of viral structural and non-structural proteins are synthesized and processed primarily in the ER. To facilitate this process, coronaviruses have developed strategies to modulate signal transduction pathways that govern ER function. We and others have previously shown that severe acute respiratory syndrome coronavirus (SARS-CoV) and mouse hepatitis virus (MHV) spike (S) proteins induce ER stress and activate cellular unfolded protein response (UPR) in the ER [3-5]. Several other viral proteins of SARS-CoV are also known to be capable of activating the UPR [6-9].

Coronaviruses that are known to infect humans broadly include two categories of viruses. In the first category, the viruses commonly circulate in human populations and cause generally mild respiratory illnesses. These viruses that are thought to be well adapted to humans include human coronavirus 229E (HCoV-229E), HCoV-OC43, HCoV-NL63 and HCoV-HKU1 [10-12]. In the family *Coronaviridae*, HCoV-229E and HCoV-NL63 belong to the genus *Alphacoronavirus*, whereas HCoV-OC43 and HCoV-HKU1 are in the lineage A of the genus *Betacoronavirus*. In the second category, the viruses accidentally or newly cross the species barrier to infect humans. SARS-CoV and the emerging Middle East respiratory syndrome coronavirus (MERS-CoV) are two well known examples in this category [13-16]. They are less well adapted to humans and cause severe and highly lethal diseases. Whereas SARS-CoV belongs to lineage B of the genus *Betacoronavirus*, MERS-CoV represents lineage C of the same genus. The molecular mechanisms that determine the severity of diseases in coronavirus infection remain poorly understood. Particularly, it is not known whether S proteins of human coronaviruses in the above two categories have similar UPR-activating activity. We therefore set out to compare S proteins of SARS-CoV and HCoV-HKU1 for their ability to modulate the UPR. All three branches of the UPR, which are governed by ER-resident transmembrane proteins ATF6, IRE1 and PERK, respectively [17,18], were examined.

HCoV-HKU1 is a betacoronavirus initially identified in 2005 from a patient with community-acquired pneumonia [11]. It was subsequently found to be commonly associated with respiratory tract infections worldwide [12,19,20]. However, molecular and cellular pathogenesis of HCoV-HKU1 in contrast to SARS-CoV remains elusive. Although primary human ciliated airway epithelial cells and type II alveolar epithelial cells have been tested for culturing of HCoV-HKU1 with limited success [21-23], HCoV-HKU1 remains a very-difficult-to-culture virus in most laboratories. Worse still, an infectious clone of HCoV-HKU1 is not available. At this stage, analysis of cloned HCoV-HKU1 genes represents a major route for deriving mechanistic insight on HCoV-HKU1 pathogenesis.

Coronavirus S proteins mediate the interaction with host cell receptors, membrane fusion and the induction of humoral and cellular immune responses [1,24]. In addition, S proteins play an important role in coronaviral pathogenesis by modulating host protein synthesis, cytokine secretion and stress response [4,5,25-27]. Changes in S proteins are critical determinants in cross-species transmission [28]. In this context, comparison of the UPR-activating activity of SARS-CoV and HCoV-HKU1 S proteins might shed new light on their roles in coronavirus-host interaction.

In this study, we provided the first evidence for the activation of ER stress and the UPR by HCoV-HKU1 S protein. We compared the UPR-activating activity of SARS-CoV and HCoV-HKU1 S proteins in terms of their influence on the expression of UPR effectors Grp78, Grp94, CHOP and PERK. We also assessed the impact of N-linked glycosylation on the activation of UPR signaling by S proteins. Furthermore, we defined a minimal domain in S1 subunit required for the UPR-activating activity of SARS-CoV S protein. Our findings provide new molecular details for UPR activation by SARS-CoV and HCoV-HKU1 S proteins.

## Results

### Expression, localization and cleavage of SARS-CoV and HCoV-HKU1 S proteins

**Figure 1:**
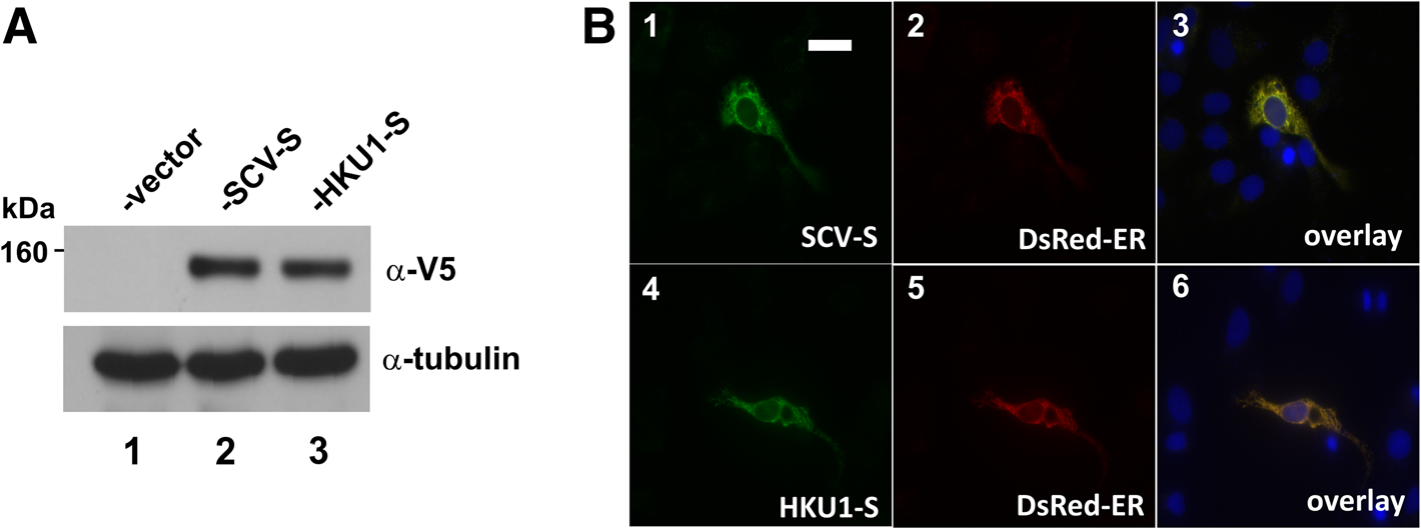
**Expression and localization of SARS-CoV and HCoV-HKU1 S proteins. (A)** Western blot analysis. 293FT cells were transfected with pLenti-SCV-S and pLenti-HKU1-S constructs expressing V5-tagged SARS-CoV S protein (SCV-S) and HCoV-HKU1 S protein (HKU1-S), respectively. Cells in the control group received empty p-Lenti vector only. Cells were lysed and immunoblotted with anti-V5 and anti-α-tubulin antibodies. **(B)** Confocal immunostaining. SARS-CoV and HCoV-HKU1 S proteins were expressed in HeLa cells and stained with anti-V5 antibody (panels 1 and 4). DsRed-ER was used as an ER marker (panels 2 and 5). The S (green) and DsRed-ER (red) fluorescent signals are overlaid and colocalization is in yellow (panels 3 and 6). Nuclear morphology was visualized with DAPI and is in blue. Bar, 20 μM.

Before we compared the UPR-activating activity of SARS-CoV and HCoV-HKU1 S proteins, we expressed them in 293FT cells. Both Western blotting (Figure 1A) and confocal immunofluorescence microscopy (Figure 1B) indicated effective expression of both proteins to similar levels. The molecular sizes of both S proteins were ~155 kDa, indicative of post-translational modifications. This was generally consistent with the reported size of SARS-CoV S protein [2-4]. With the help of the fluorescent ER marker of DsRed-ER, we confirmed that both S proteins resided largely in the ER (Figure 1B, panels 3 and 6).

**Figure 2:**
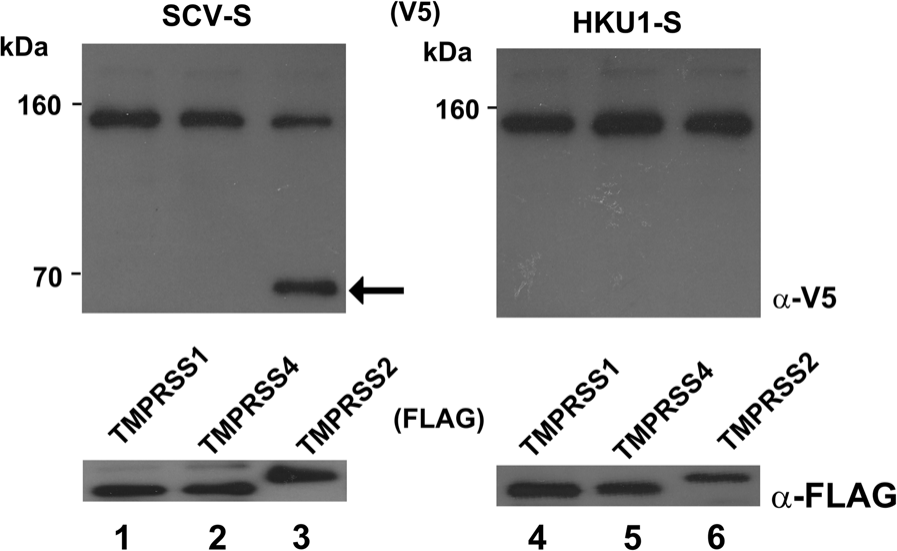
**Proteolytic cleavage of SARS-CoV S protein by TMPRSS2.** 293FT cells were cotransfected with FLAG-tagged TMPRSS1/4/2 and V5-tagged S proteins. Western blotting was performed with anti-V5 and anti-FLAG antibodies. Arrow points to S1 subunit of SARS-CoV.

SARS-CoV S protein is proteolytically processed into S1 and S2 subunits by host proteases such as cathepsin L, factor Xa, trypsin and transmembrane serine protease TMPRSS2 [29-34]. Particularly, TMPRSS2 efficiently activates S proteins of SARS-CoV as well as MERS-CoV and HCoV-229E [32-36]. Moreover, TMPRSS2 and related proteases TMPRSS4, which are abundantly expressed in human alveolar epithelial cells, are capable of activating influenza virus hemagglutinin [37]. To investigate whether they might also proteolytically activate HCoV-HKU1 S protein, we expressed them together in 293FT cells. Coexpression of TMPRSS2 with SARS-CoV S protein resulted in the appearance of S1 subunit, indicative of proteolytic cleavage (Figure 2, lane 3). Although a V5 tag was added to the N-terminus of SARS-CoV S protein, the cleavage was not affected. The action of TMPRSS2 on SARS-CoV S was highly specific since TMPRSS1 and TMPRSS4 had no activity under the same condition (Figure 2, lanes 1 and 2). In contrast, none of the three proteases cleaved HCoV-HKU1 S protein (Figure 2, lanes 4–6). Thus, HCoV-HKU1 S protein is not processed by TMPRSS1, TMPRSS2 or TMPRSS4.

### S proteins from both SARS-CoV and HCoV-HKU1 activate UPR

**Figure 3:**
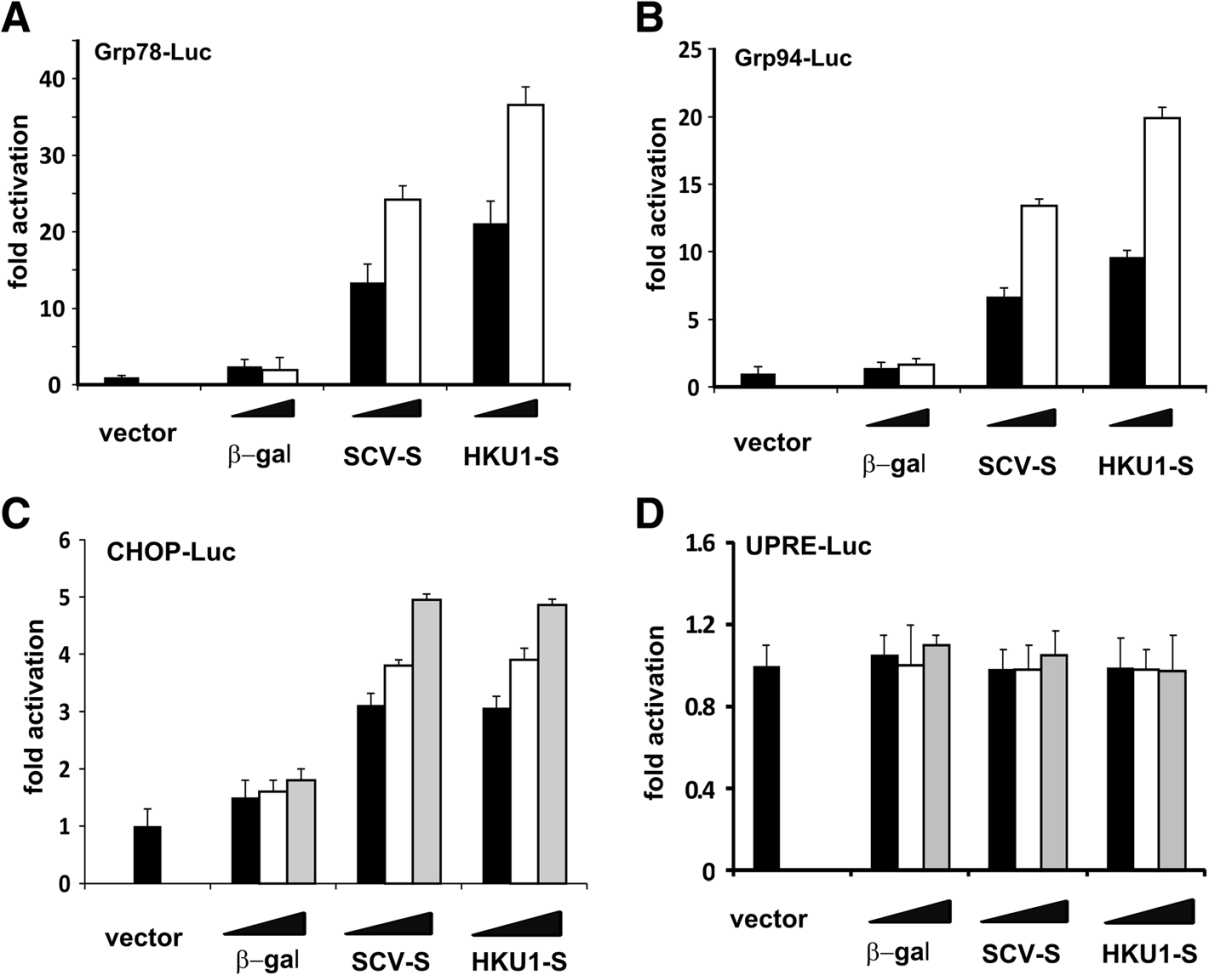
**UPR activation by S proteins.** 293FT cells were transfected with the indicated expression vector together with luciferase reporter plasmid pGrp78-Luc **(A)**, pGrp94-Luc **(B)**, pCHOP-Luc **(C)** or pUPRE-Luc **(D)**. Cells were harvested 36 h post-transfection for dual luciferase assay. Progressively escalating amounts of expression plasmids for β-galactosidase (β-gal) and S proteins were used. Fold activation was calculated from readouts of firefly luciferase activity normalized to those of *Renilla* luciferase activity. Activity recovered from cells transfected with pLenti vector alone was set as 1. Means from triplicate experiments are presented and error bars indicate SD. In panel **D**, there is no statistically significant difference between groups β-gal and SCV-S or between groups β-gal and HKU1-S (p > 0.05).

We and others have previously demonstrated transcriptional activation of Grp78 and Grp94 promoters by SARS-CoV S protein [3-5]. Grp78 and Grp94 are molecular chaperones that are drastically induced in response to ER stress. They therefore served as robust indicators of the UPR [17,18]. The promoter activity of Grp78 and Grp94 was activated by S proteins of SARS-CoV and HCoV-HKU1 to similar levels (Figure 3A and B). The stimulatory effect of HCoV-HKU1 S protein is dose-dependent and equally potent compared to that of SARS-CoV S protein. In contrast, overexpression of β-galactosidase, a large foreign protein, had minimal or very mild effect on Grp78 and Grp94 promoter activity. Thus, S proteins from both viruses might induce ER stress and activate the UPR.

We next tested the influence of S proteins on the transcriptional activity driven by CHOP promoter and UPRE. CHOP is a proapoptotic transcription factor activated in the UPR [38,39]. UPRE is an enhancer element responsive to ATF6, XBP1 and CREB3-related transcription factors [40-43]. UPRE mediates the activation of a subset of UPR genes distinct from Grp78 and Grp94 [44,45]. S proteins of both viruses exhibited the same activity profile not only on Grp78 and Grp94 promoters, but also on CHOP promoter and UPRE. They activated CHOP promoter mildly but did not affect the activity of UPRE (Figure 3C and D). Thus, S proteins of SARS-CoV and HCoV-HKU1 showed the same pattern of modulatory activity on UPR effector genes.

**Figure 4:**
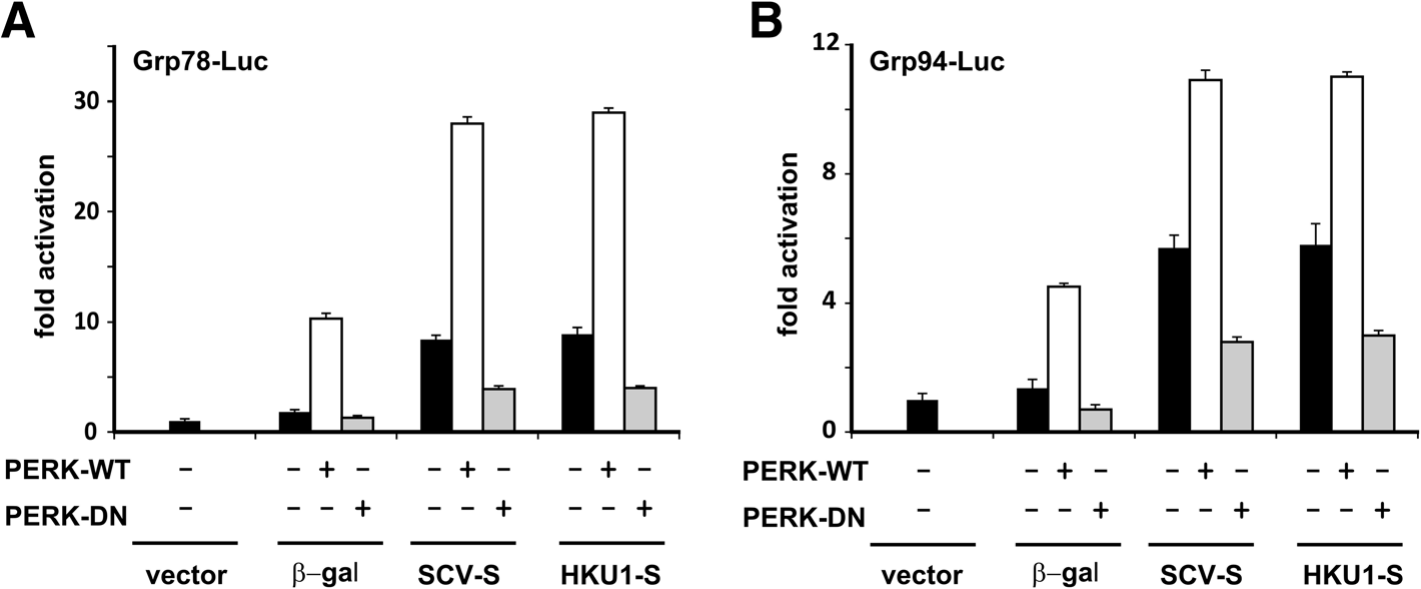
**Requirement of PERK for UPR activation by S proteins.** 293FT cells were transfected with the indicated expression vectors together with either pGrp78-Luc **(A)** or pGrp94-Luc **(B)** reporter plasmid. Cells were harvested 36 h post-transfection for dual luciferase assay.

PERK is a protein kinase that controls the expression of many UPR effector genes including Grp78 and Grp94 [17,46,47]. To analyze further whether PERK activity is required for transcriptional activation of Grp78 and Grp94 promoters by SARS-CoV and HCoV-HKU1 S proteins, we made use of a dominant negative (DN) mutant of PERK which constitutively inhibits PERK kinase activity [48]. If the activation of Grp78 and Grp94 proteins by S proteins requires PERK, their stimulatory effect would be reversed in the presence of PERK-DN. Consistent with our previous findings [3], activation of Grp78 and Grp94 promoters by SARS-CoV S protein was enhanced by wild-type PERK (PERK-WT) and dampened by PERK-DN (Figure 4A and B). A very similar pattern was also observed for HCoV-HKU1 S protein. In other words, PERK-WT and PERK-DN exerted opposite effects on the activation of Grp78 and Grp94 promoters by S proteins from both viruses (Figure 4A and B). Thus, the activation of Grp78 and Grp94 expression by S proteins is mediated through PERK.

### N-linked glycosylation is not required for UPR activation by S proteins

**Figure 5:**
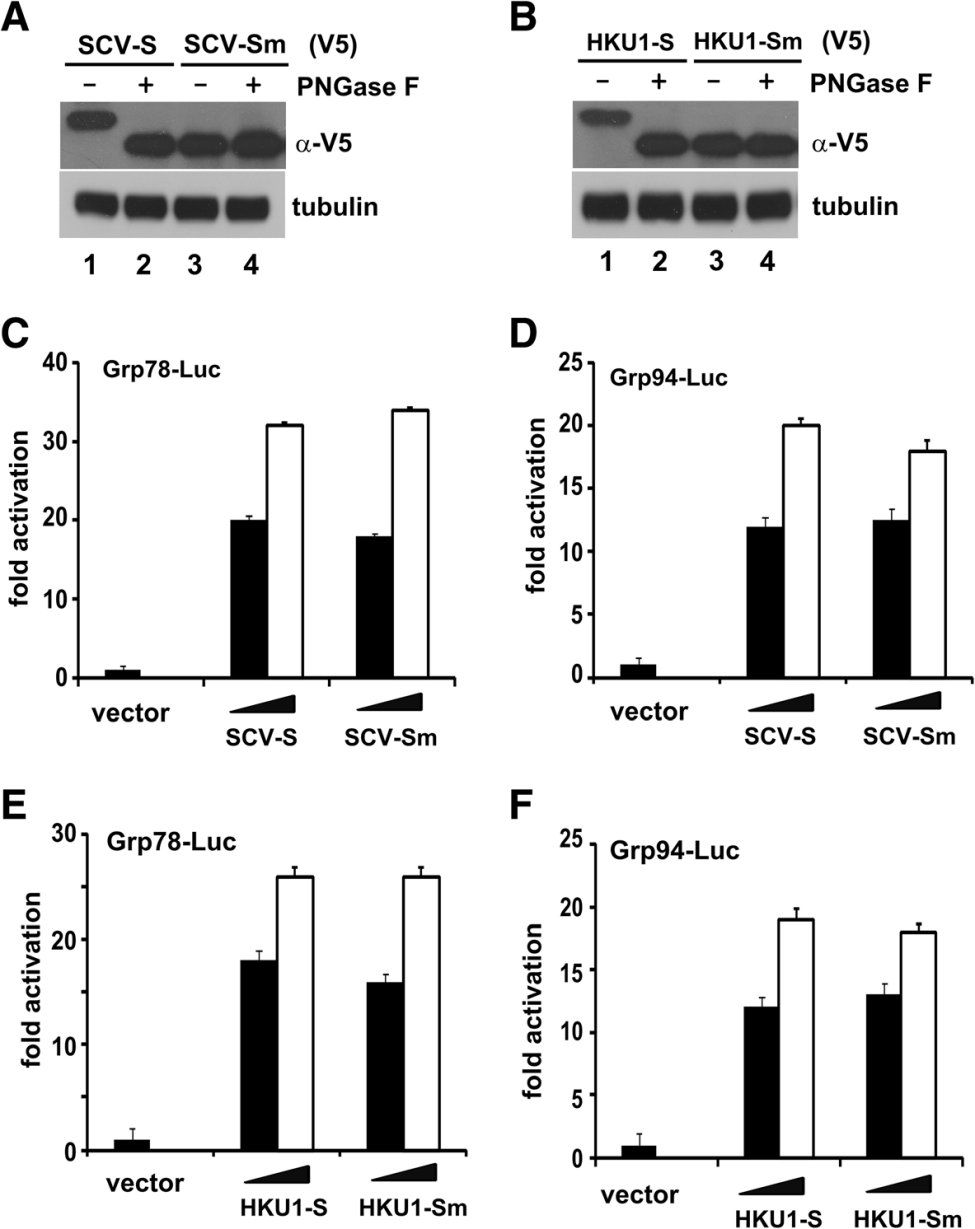
**Impact of N-linked glycosylation on UPR activation by S proteins. (A, B)** N-linked glycosylation mutants. SARS-CoV and HCoV-HKU1 S proteins and their N-linked glycosylation mutants (SCV-Sm and HKU1-Sm) were expressed in 293FT cells. To remove N-linked glycans, cell lysates were incubated with endoglycosidase PNGase F for 1 h at 37°C. **(C-F)** Luciferase reporter assay. S proteins and their N-linked glycosylation mutants in escalating dose were compared for the activity to activate luciferase reporter expression driven by Grp78 and Grp94 promoters.

Glycosylation might affect the folding, stability, sorting and function of viral structural proteins [49]. Coronavirus S protein is heavily glycosylated. N-linked glycosylation of SARS-CoV S protein is known to be critical to receptor binding, viral entry and infectivity [50-52]. Since N-linked glycosylation of S protein might overload the ER leading to the activation of the UPR, it will be of interest to see whether N-linked glycosylation of SARS-CoV and HCoV-HKU1 S proteins might be influential in their induction of ER stress. N-linked glycosylation sites in SARS-CoV S protein has been documented [50-52]. Based on comparison and prediction with the help of a computer program, ten major N-linked glycosylation sites in SARS-CoV S protein and nine sites in HCoV-HKU1 S protein were chosen for further analysis. When all these sites were mutated, electrophoretic mobility of the mutant S proteins on SDS-PAGE gel was no long shifted upon treatment of cell lysates with endoglycosidase PNGase F (Figure 5A and B), indicating their deficiency in N-linked glycosylation. However, compared to the wild-type proteins, these mutants were equally competent in the activation of Grp78 and Grp94 promoters (Figure 5C-F). Hence, N-linked glycosylation is not influential in UPR activation by SARS-CoV and HCoV-HKU1 S proteins.

### Mapping of UPR-activating domain in SARS-CoV S protein

**Figure 6:**
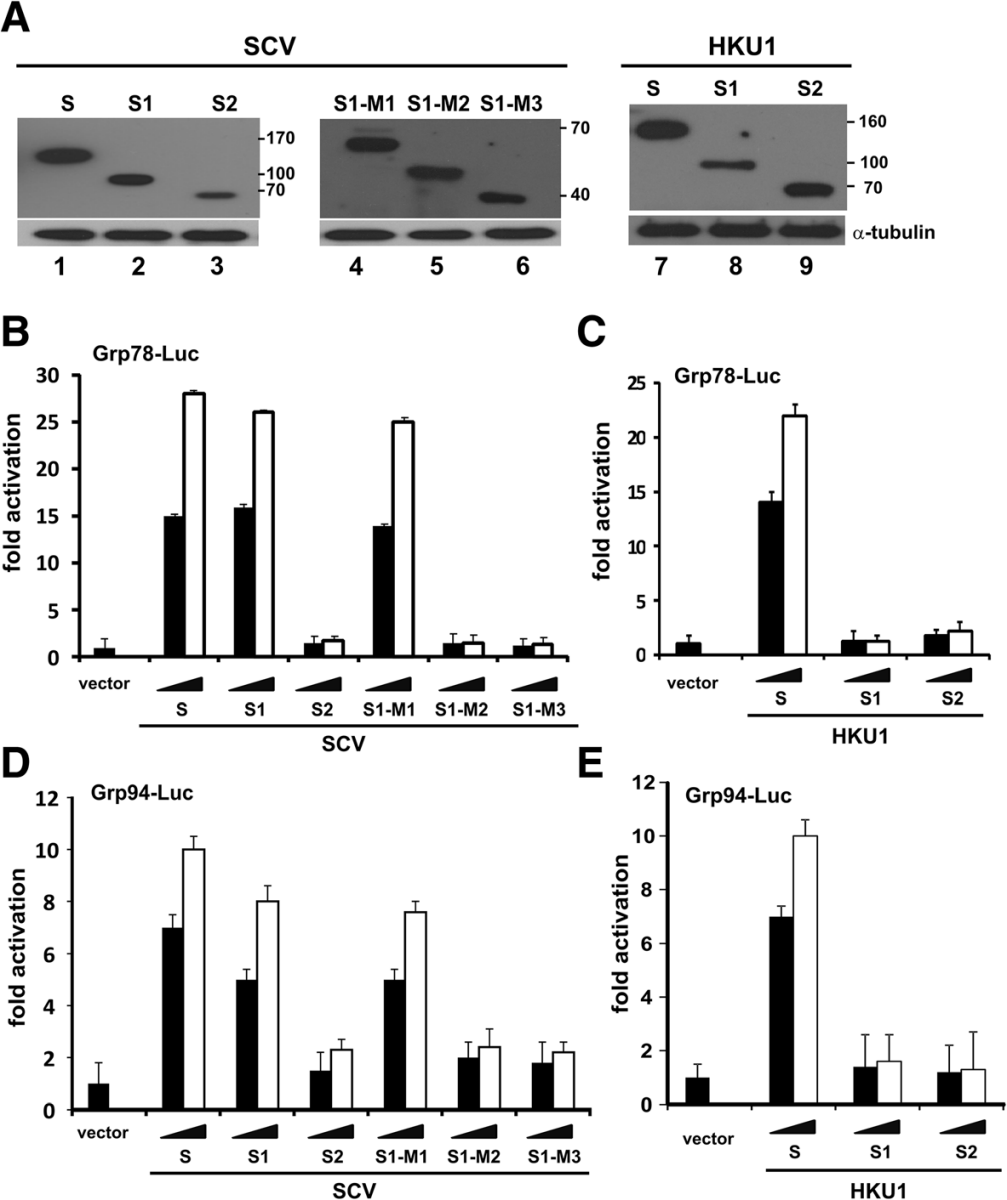
**Mapping of UPR-activating domain in SARS-CoV and HCoV-HKU1 S proteins. (A)** Truncated mutants. SARS-CoV (SCV) S protein and its truncated mutants S1 (amino acids 1–770), S2 (amino acids 771–1255), S1-M1 (amino acids 201–770), S1-M2 (amino acids 401–770) and S1-M3 (amino acids 534–770) as well as HCoV-HKU1 S protein and its truncated mutants S1 (amino acids 1–869) and S2 (amino acids 870–1356) were expressed in 293FT cells. **(B-E)** Luciferase reporter assay. S protein and its truncated mutants in escalating doses were compared for the activity to activate luciferase reporter expression driven by Grp78 and Grp94 promoters.

SARS-CoV S protein is cleaved into S1 and S2 subunits [29-34]. Above we showed the proteolytic cleavage of SARS-CoV S protein by TMPRSS2 protease (Figure 2), but the processing of HCoV-HKU1 S protein is not mediated by this enzyme and remains elusive. To determine whether the UPR-activating property of SARS-CoV S protein is mediated by S1 (amino acids 1–770) or S2 (amino acids 771–1255) subunit, we expressed them in 293FT cells (Figure 6A, lanes 2 and 3). Whereas S2 has no influence on the activation of Grp78 and Grp94 promoters, S1 was fully competent in this activation (Figure 6B and D). To compare the two S proteins, we also expressed the polypeptides corresponding to S1 (amino acids 1–869) and S2 (amino acids 870–1255) of HCoV-HKU1 (Figure 6A, lanes 8 and 9), although we had no evidence in support of the cleavage of S in that virus. To our surprise, neither S1 nor S2 of HCoV-HKU1 was able to activate Grp78 or Grp94 promoter (Figure 6C and E).

To further dissect the UPR-activating domain in SARS-CoV S1 subunit, we constructed three truncated mutants S1-M1 (amino acids 201–770), S1-M2 (amino acids 401–770) and S1-M3 (amino acids 534–770). These mutants were expressed in 293FT cells (Figure 6A, lanes 4–6). Among them only S1-M1 was capable of activating the transcriptional activity of Grp78 and Grp94 promoters (Figure 6B and D). Neither S1-M2 nor S1-M3 was active in the same assay. Thus, the central region (amino acids 201–400) of SARS-CoV S1 subunit is indispensable for the UPR-activating activity.

## Discussion

In this study we compared and contrasted the UPR-activating activity of S proteins of SARS-CoV and HCoV-HKU1. We found that the two S proteins share the following three properties in common. First, they localize predominantly to the ER (Figure 1). Second, they display a similar profile of UPR-activating properties with the ability to activate Grp78, Grp94 and CHOP promoters but not UPRE enhancer (Figure 3). Third, their activation of Grp78 and Grp94 promoters requires catalytic activity of PERK (Figure 4) but not N-linked glycosylation (Figure 5). On the other hand, the two S proteins also exhibit distinct properties in protease cleavability and UPR-activating domain. TMPRSS2 protease is capable of cleaving SARS-CoV S protein into S1 and S2 subunits, but has no proteolytic activity on HCoV-HKU1 S protein (Figure 2). The S1 subunit of SARS-CoV sufficiently activates the UPR, but its counterpart in HCoV-HKU1 has no UPR-modulating activity (Figure 6). Thus, although the UPR-activating domains in SARS-CoV and HCoV-HKU1 S proteins are distinct, their modulatory effects on UPR signaling are similar.

We provided the first evidence for the ability of HCoV-HKU1 S protein to modulate the UPR. This adds HCoV-HKU1 to the list of coronaviruses including SARS-CoV and MHV, which use S protein to activate the UPR [3-5]. Although the direct evidence remains to be seen, HCoV-OC43, another human betacoronavirus of lineage A, might also use S protein to modulate the UPR, since a mutant HCoV-OC43 carrying two persistence-associated mutations in S was able to activate the UPR more potently [53]. It will be of interest to see whether other coronaviruses including the emerging MERS-CoV might also employ S protein to activate the UPR. More importantly, new investigations should be directed towards understanding the biological significance of UPR activation in coronavirus life cycle.

Whereas the UPR-activating domain maps to a central region (amino acids 201–400) in SARS-CoV S protein, the S1 fragment (amino acids 1–869) of HCoV-HKU1 S protein was unable to activate the UPR (Figure 6). The central regions of the two S proteins are relatively less conserved. It remains to be seen whether HCoV-HKU1 S protein might use one part of S1 and another part of S2 to perform its function in UPR modulation. Another possibility is that some regions in the S1 fragment of HCoV-HKU1 could exert suppressive effect on UPR activation. Further experiments are required to define the UPR-activating domain in HCoV-HKU1 S protein.

Elevated expression of molecular chaperones such as Grp78 and Grp94 would plausibly increase the capacity of ER to fold and process coronaviral proteins produced in extraordinarily high amounts during viral replication. This might explain why Grp78 and Grp94 promoters are activated potently by S proteins. On the other hand, CHOP mediates ER stress-induced apoptosis and UPRE controls the transcription of some UPR effector genes involved in ER-associated protein degradation, such as EDEM [38,45]. Compared to Grp78 and Grp94 promoters, the activation of CHOP promoter by S proteins was very modest (Figure 3C). Moreover, S proteins did not activate UPRE-dependent transcription (Figure 3D). Hence, UPR activation by S proteins is highly selective and in the benefit of the viruses. This is in line with the idea that ER stress-induced apoptosis or ER-associated protein degradation would be undesirable in the early phase of SARS-CoV and HCoV-HKU1 replication. Exactly how S proteins differentially modulate UPR signaling to facilitate viral replication merits further analysis.

HCoV-HKU1 remains unculturable except in primary human airway or alveolar epithelial cells [21-23]. This and the lack of an infectious HCoV-HKU1 clone prevented us from analyzing UPR activation in infected cells. Establishing a more accessible and efficient culture system and an animal model for the study of HCoV-HKU1 infection is the next challenge in the field. In addition, a recombinant lentivirus pseudotyped with HCoV-HKU1 S protein can also be used to study the roles of S protein in viral entry and pathogenesis. Particularly, such a pseudotyped virus might prove useful in the analysis of UPR activation by HCoV-HKU1 S protein.

SARS-CoV is a highly pathogenic coronavirus in humans, whereas human infection with HCoV-HKU1 is more common but causes less severe disease [12,19,20]. Because S proteins from both viruses are equally competent in the activation of the UPR, the UPR-modulating property of S proteins is unlikely a critical determinant in the severity of disease associated with SARS-CoV and HCoV-HKU1. However, TMPRSS2 protease was capable of cleaving SARS-CoV S protein, but not HCoV-HKU1 S protein, into S1 and S1 subunits (Figure 2). Moreover, SARS-CoV S1 protein, but not its counterpart in HCoV-HKU1, was required and sufficient for UPR activation (Figure 6). Cleavability of surface proteins by host proteases is an important virulence determinant in coronaviruses and other viruses such as influenza [1,14,37]. In this connection, it will not be too surprising if the inability of TMPRSS2 to cleave HCoV-HKU1 might affect pathogenesis. It will be even more interesting to see whether the ability of S1 to activate the UPR might be related to viral replication and pathogenesis.

Our findings that SARS-CoV and HCoV-HKU1 S proteins activate ER stress and the UPR might have implications in therapeutic intervention. Pharmaceutical modulators of ER stress and the UPR have been developed and tested for various disease conditions including viral infection [54,55]. Interestingly, whereas inhibition of PERK kinase has been found to inhibit cytomegalovirus replication [56], activation of the UPR with a small-molecule compound also has broad-spectrum antiviral activity [57]. Thus, our demonstration of the activation of the UPR by S proteins might pave the way for further evaluation of the utility of UPR-modulating agents for the treatment of diseases associated with SARS-CoV and HCoV-HKU1 infection.

## Materials and methods

### Plasmids and antibodies

Expression plasmids for human PERK and its DN mutant K621M were obtained from Ronald Wek [48]. Reporter plasmid pCHOP-Luc, in which luciferase expression is driven by human CHOP promoter (-644 to +91), was provided by Nai Sum Wong [23]. Reporter plasmids pGRP78-Luc and pGRP94-Luc were gifts from Kazutoshi Mori [40,58]. The Grp78 and Grp94 promoters are derived from -304 to +34 of human Grp78 gene and -363 to +34 of human Grp94 gene, respectively. Both promoters harbor multiple copies of ER stress response element [58]. pUPRE-Luc reporter plasmid has been described elsewhere [41,42].

Mouse monoclonal anti-V5 antibody was purchased from Invitrogen. Mouse anti-FLAG antibody (clone M2) was from Sigma-Aldrich.

### Cell culture and transfection

293FT and HeLa cells were grown in Dulbecco’s modified Eagle’s medium containing 10% fetal bovine serum and antibiotics. Cells were transfected with Gene-Juice transfection reagent (Novagen) as described [42,59]. The SV40 large T antigen is constitutively expressed in 293FT cells.

### Western blotting and luciferase reporter assay

Western blotting and dual luciferase reporter assay were carried out as described [42,59]. Control plasmid pRLSV40 expressing *Renilla* luciferase (Promega) was cotransfected into cells and firefly luciferase activity was normalized to that of *Renilla* luciferase in all experiments.

### Laser-scanning confocal microscopy

Confocal immunofluorescence microscopy was performed on LSM510 (Carl-Zeiss) as described [60,61]. HeLa cells were transfected with an S-expressing plasmid and pDsRed-ER (Clontech) for 36 h. Cells were then fixed and stained with anti-V5 antibody. Nuclei were counter-stained with 4', 6-diamidino-2-phenylindole (DAPI) before mounting.

### Site-directed mutagenesis

N-linked glycosylation mutants of S proteins were constructed by using a site-directed mutagenesis kit (Agilent). Potential N-linked glycosylation sites were analyzed online by the NetNGlyc program (website: http://www.cbs.dtu.dk/services/NetNGlyc/). For SARS-CoV S protein, asparagine residues at positions 29, 65, 119, 227, 318, 330, 357, 589, 669 and 783 were mutated to glutamine. For HCoV-HKU1 S protein, asparagine residues at positions 19, 29, 192, 335, 433, 454, 664, 684 and 725 were mutated to glutamine.

### Endoglycosidase treatment

N-linked glycans were removed from S protein by endoglycosidase treatment as described [42]. In brief, cell lysates were incubated with endoglycosidase PNGase F (New England BioLabs) for 1 h at 37°C.

## Competing interests

The authors declare that they have no competing interests.

## Authors’ contributions

KLS, KHK, PCYW and DYJ designed research and analyzed data. KLS and CPC performed research. KLS and DYJ wrote the paper. All authors read and approved the final manuscript.
